# Disseminated Mpox infection, a rare case of an immunocompromised patient treated with tecovirimat and brincidofovir

**DOI:** 10.1016/j.jdcr.2025.02.002

**Published:** 2025-03-05

**Authors:** Paytra A. Klein, Rose Parisi, Ananthakrishnan Ramani

**Affiliations:** a Albany Medical College, Albany, New York; b Department of Dermatology, Warren Alpert Medical School of Brown University, Providence, Rhode Island; c Department of Internal Medicine, Albany Medical Health System, Albany, New York; d Division of Infectious Disease, Albany Medical Health System, Albany, New York

**Keywords:** AIDS, brincidofovir, HIV, Mpox, tecovirimat

## Introduction

The Mpox virus (MPXV) is a member of the Orthopoxvirus genus in the Poxviridae family.^1^ Mpox is a zoonotic disease, caused by MPXV, which is characterized by a flu-like prodrome, lymphadenopathy, and a rash. The rash is often composed of centrifugally spreading papules that progress to umbilicated pustules, which ulcerate, crust, and then re-epithelialize.^1^ Mpox can be transmitted through exposure to infected animals, direct contact with infected humans, fomites, or respiratory droplets.^2^

MPXV has 2 clinically indistinguishable types: clade I and clade II.^3^ Clade I is endemic to Central Africa,^4^ while clade II is endemic to West Africa.^5^ In July 2022, the multicountry outbreak of clade II was declared a Public Health Emergency of International Concern by the World Health Organization.^6^ In August 2024, the World Health Organization declared the clade I Mpox outbreak in Central and Eastern Africa a Public Health Emergency of International Concern.^4^^,^^6^

There are no approved treatments for Mpox. However, clinical trials are investigating the use of smallpox antivirals tecovirimat (TPOXX) and brincidofovir (Tembexa)^7^; including the National Institute of Allergy and Infectious Disease Study of Tecovirimat for Human Mpox Virus (STOMP) trial^7^ and the MpOx Study in Africa trial.^8^ We report a complex case of a patient with HIV/AIDS who presented with disseminated Mpox clade II that initially showed an incomplete response to tecovirimat alone but improved significantly with combination therapy using both tecovirimat and brincidofovir.

## Case report

**Fig 1:**
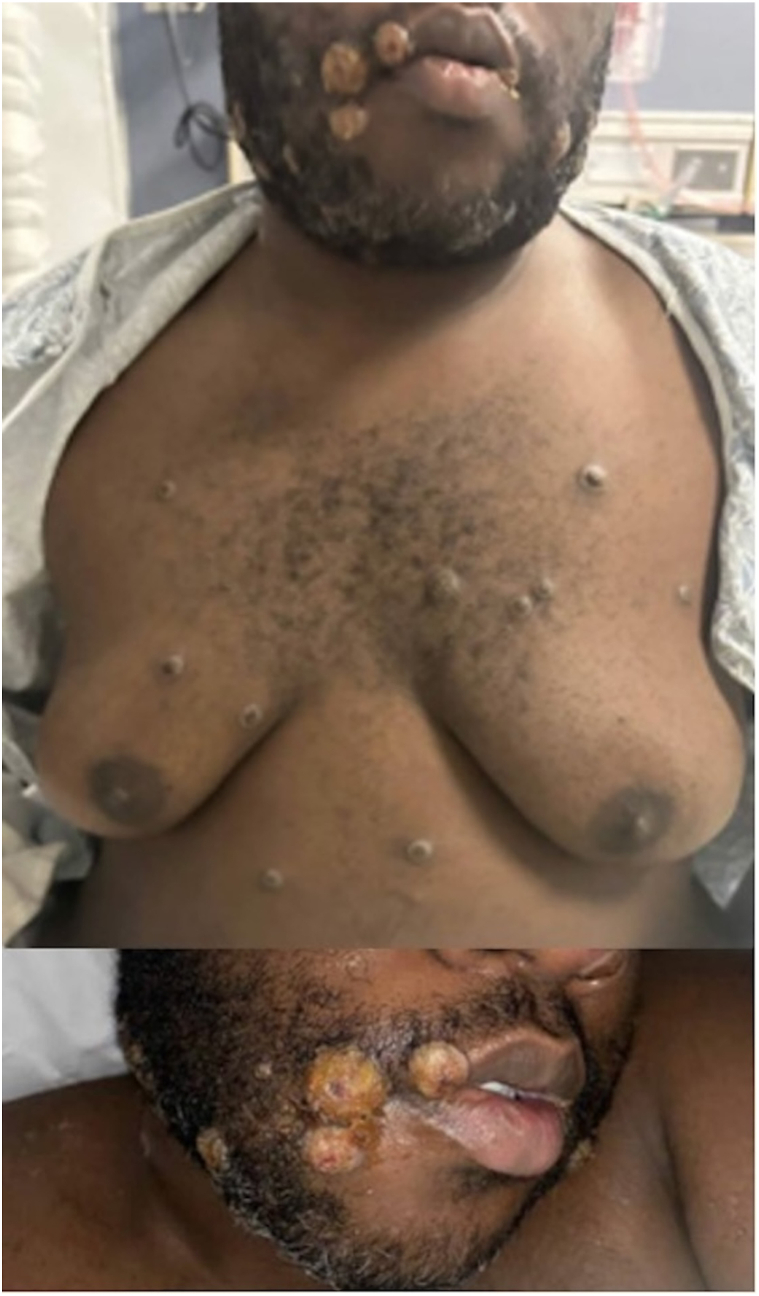
Initial presentation of Mpox lesions.

A 38-year-old transgender male-to-female with a history of poorly controlled HIV/AIDS (not on antiretroviral therapy) and recently treated syphilis presented with intermittent fevers and a 9-day history of a worsening rash. The rash began on the face and perianal area and then spread to the extremities, chest, trunk, and back. Examination revealed diffuse vesicles, pustules, crusted ulcerated papules with serosanguinous drainage, and excoriations (Fig 1). The palms and soles were spared. Perianal lesions coalesced into ulcerated papules, while facial lesions were blood-tinged and draining.

The patient reported being sexually active with one male partner, endorsed consistent condom use, and denied drug use. The patient was monogamous but believed her partner was not. It is unknown whether her partner had lesions as our patient did not maintain communication; he was the suspected source of infection. CD4 count was 175 cells/μL and HIV-1 RNA was 11,420 copies/mL. Chlamydia trachomatis, Neisseria gonorrhoeae, Herpes simplex virus, and Hepatitis C were negative. Treponema Pallidum Antibody was reactive, but rapid plasma reagin was negative. A skin swab tested positive for Mpox DNA by polymerase chain reaction (PCR).

**Fig 2:**
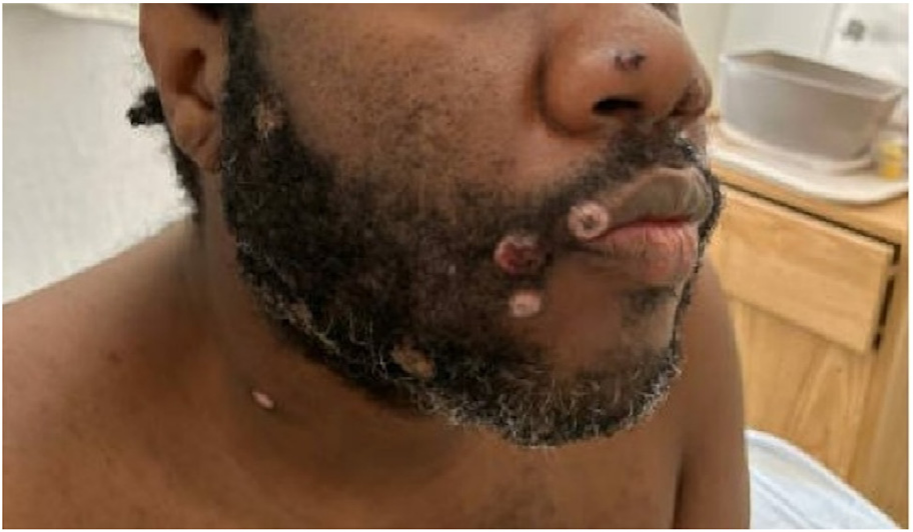
Mpox lesions in healing phase.

The patient was enrolled in the National Institute of Allergy and Infectious Disease STOMP trial and completed a 14-day course of tecovirimat at a dose of 600 mg twice daily. The patient’s only reported side effect was headache on day 2 of treatment. The patient’s lesions substantially improved (Fig 2). She was discharged on antiretroviral therapy (dolutegravir/emtricitabine-tenofovir) and prophylactic Bactrim and followed-up outpatient at the HIV Clinic.

**Fig 3:**
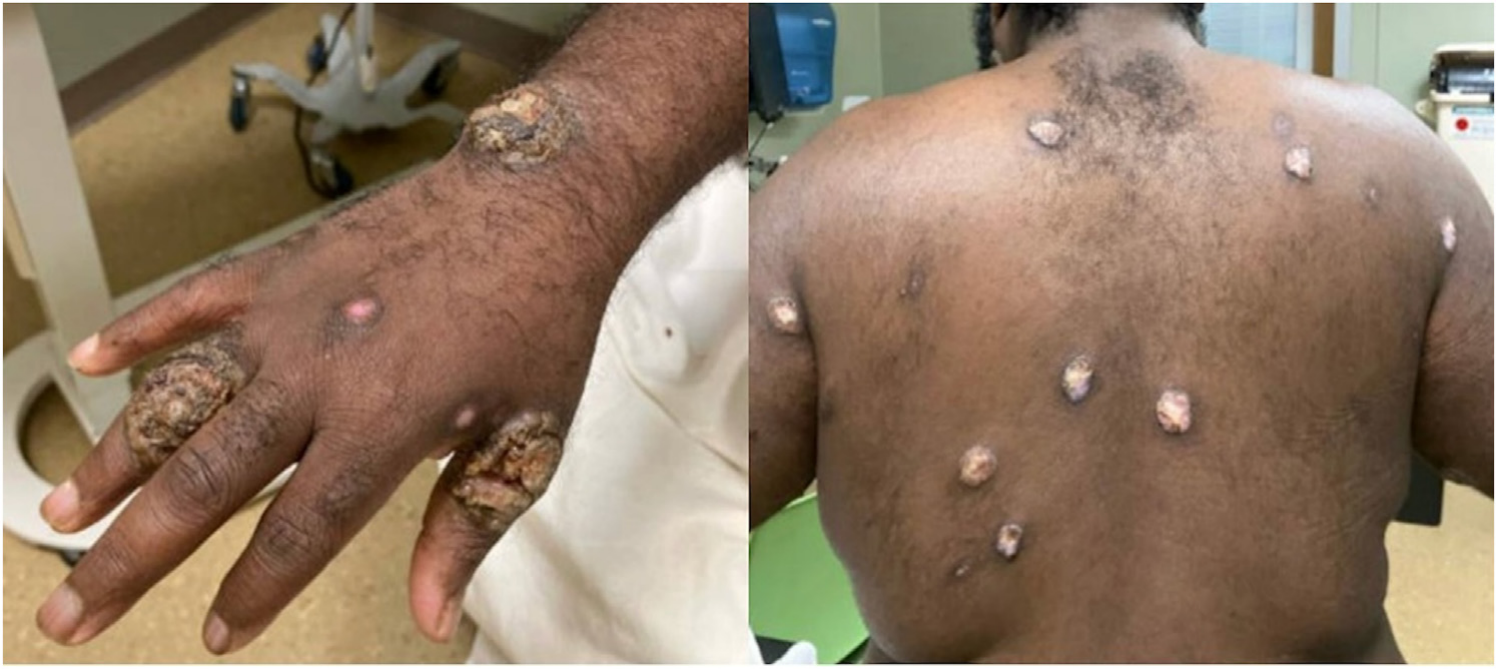
Recurrent and worsening Mpox lesions.

**Fig 4:**
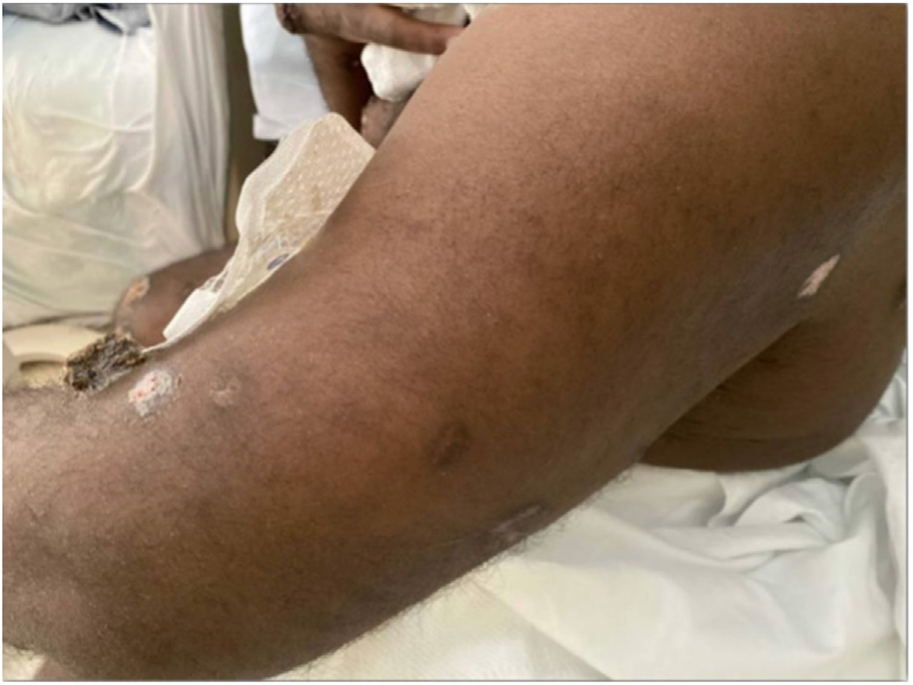
Mpox lesions in healing phase.

However, about one and a half months later, the patient was readmitted with worsening Mpox lesions characterized by increased drainage and severe pain, new facial and limb lesions and 4 days of intermittent fever (Fig 3). Mpox DNA skin PCR was again positive. Both MPXV Clade II DNA and Non-Variola OPXV DNA were detected by real-time PCR. Patient had not received the Mpox vaccine. She declined skin biopsy and culture. Empiric treatment for presumed superimposed bacterial infection was initiated with linezolid and piperacillin-tazobactam for 5 days. The patient’s severe pain was managed with multimodal therapy. After discussions about this complicated patient with the Centers for Disease Control and Prevention, Food and Drug Administration and the STOMP trial team, the patient was started on an extended 28-day course of Tecovirimat (600 mg BID) and Brincidofovir (200 mg weekly, 4 doses). Wound care included gentle cleansing with soap and water, topical white petrolatum, and nonstick gauze dressings. Over time, no new lesions developed, and existing ones healed (Fig 4). She was discharged after a 1-month hospitalization.

## Discussion

In patients with advanced HIV, Mpox can lead to more severe outcomes, including prolonged illness, widespread disseminated skin lesions, systemic complications such as secondary infections or sepsis, and higher mortality.^9^ Evidence suggests severe, fulminant, and necrotizing Mpox may be an AIDS-defining condition.^9^ Although there is no specific treatment for Mpox, the Centers for Disease Control and Prevention recommends using tecovirimat or brincidofovir to treat Mpox in severely immunocompromised patients.^7^

We report a challenging case of a 38-year-old transgender woman with HIV/AIDS who presented with disseminated cutaneous Mpox clade II. The initial 14-day course of tecovirimat alone yielded an incomplete response. However, significant improvement was achieved with a combination of an extended 28-day course of tecovirimat and brincidofovir. Our patient’s advanced HIV and immunocompromised status likely contributed to her partial response to initial treatment. The recurrence of lesions was thought to be due to a combination of tecovirimat resistance and immune reconstitution inflammatory syndrome. This case highlights how an extended or intensified treatment approach, including combination therapy, may be needed to effectively manage Mpox in severely immunocompromised patients.

Notably, our patient experienced 1 day of headache during the initial 14-day course of tecovirimat, which is a known side effect of the medication. This highlights the importance of closely monitoring for potential side effects when using investigational drugs.

Beyond antivirals, wound care is essential for Mpox management and includes daily gentle cleansing of lesions with soap and water, petroleum application, and use of gauze dressings.^10^ Pain, often disproportionate to lesion appearance, is common in Mpox and can be managed with non-steroidal anti-inflammatory drugs, acetaminophen, topical steroids, or topical lidocaine. For severe pain, short-term use of gabapentin or opioids may be considered, carefully balancing risks and benefits. Additionally, collaborating with the Centers for Disease Control and Prevention, Food and Drug Administration, and clinical trial teams is crucial for managing complex cases in immunocompromised patients, as it was for formulating our patient’s care plan.

In conclusion, we report a complex case of a transgender female with advanced HIV and disseminated Mpox clade II that improved with an extended use of investigative smallpox antivirals, wound care, and pain management. Further research is needed exploring the use of tecovirimat, brincidofovir among other antivirals for the treatment of Mpox.

## Conflicts of interest

None disclosed.
